# Assessment of Short- and Long-Term Mortality Displacement in Heat-Related Deaths in Brisbane, Australia, 1996–2004

**DOI:** 10.1289/ehp.1307606

**Published:** 2015-03-20

**Authors:** Zhen Qiao, Yuming Guo, Weiwei Yu, Shilu Tong

**Affiliations:** 1 School of Public Health and Social Work, and; 2 Institute of Health and Biomedical Innovation, Queensland University of Technology, Brisbane, Australia; 3 Department of Epidemiology and Biostatistics, School of Population Health, University of Queensland, Brisbane, Australia

## Abstract

**Background:**

Mortality displacement (or “harvesting”) has been identified as a key issue in the assessment of the temperature–mortality relationship. However, only a few studies have addressed the “harvesting” issue and findings have not been consistent.

**Objectives:**

We examined the potential impact of both short- and long-term harvesting effects on heat-related deaths in Brisbane, Australia.

**Methods:**

We collected data on daily counts of deaths (nonaccidental, cardiovascular, and respiratory), weather, and air pollution in Brisbane from 1 January 1996 to 30 November 2004. We estimated heat-related deaths, identified potential short-term mortality displacement, and assessed how and to what extent the impact of summer temperature on mortality was modified by mortality in the previous winter using a Poisson time-series regression combined with distributed lag nonlinear model (DLNM).

**Results:**

There were significant associations between temperature and each mortality outcome in summer. We found evidence of short-term mortality displacement for respiratory mortality, and evidence of longer-term mortality displacement for nonaccidental and cardiovascular mortality when the preceding winter’s mortality was low. The estimated heat effect on mortality was generally stronger when the preceding winter mortality level was low. For example, we estimated a 22% increase in nonaccidental mortality (95% CI: 14, 30) with a 1°C increase in mean temperature above a 28°C threshold in summers that followed a winter with low mortality, compared with 12% (95% CI: 7, 17) following a winter with high mortality. The short- and long-term mortality displacement appeared to jointly influence the assessment of heat-related deaths.

**Conclusions:**

We found evidence of both short- and long-term harvesting effects on heat-related mortality in Brisbane, Australia. Our finding may clarify temperature-related health risks and inform effective public health interventions to manage the health impacts of climate change.

**Citation:**

Qiao Z, Guo Y, Yu W, Tong S. 2015. Assessment of short- and long-term mortality displacement in heat-related deaths in Brisbane, Australia, 1996–2004. Environ Health Perspect 123:766–772; http://dx.doi.org/10.1289/ehp.1307606

## Introduction

Numerous epidemiologic studies have characterized the relationship between high temperature and mortality, including total mortality and cause-specific deaths (Baccini et al. 2008; Goodman et al. 2004; Kinney et al. 2008; McMichael et al. 2008; Medina-Ramón and Schwartz 2007; Zanobetti and Schwartz 2008). Most excess heat-related deaths have been associated with cardiovascular (CVD) and respiratory diseases, particularly among the elderly. Some heat-related deaths may occur in people whose health is already compromised, resulting in a decrease in the expected number of deaths following an initial increase, a phenomenon referred to as mortality displacement or “harvesting” (Hajat et al. 2005; Schwartz 2000). It is important to understand mortality displacement because if heat-related deaths occur only in already frail individuals with a short life expectancy, the heat impact would have less public health importance (Guo et al. 2012a; Huang et al. 2012).

Evidence of mortality displacement was first described as a short-term effect (within days or weeks) (Hajat et al. 2005). Subsequent studies reported that winter mortality levels significantly modified the estimated effect of temperature on mortality in the following summer (Ha et al. 2011; Rocklöv et al. 2009; Stafoggia et al. 2009), suggesting a process consistent with the long-term mortality displacement. However, results have not been consistent among the limited number of studies on this topic, with some reporting evidence of mortality displacement on hot days or during heat waves (Basu and Malig 2011; Hajat et al. 2005; Kyselý 2004), whereas others have reported no evidence of a short-term harvesting effect (Kyselý and Kim 2009; Le Tertre et al. 2006; Tong et al. 2010). We postulate that short- and long-term mortality displacement may jointly influence the assessment of heat-related deaths, because both affect the same pool of susceptible individuals. In this study, we assessed effects of short- and long-term harvesting on heat-related mortality in Brisbane, a subtropical city in Australia.

## Methods

*Data*. Mortality data. This study was conducted in Brisbane, the capital of Queensland, Australia. Daily counts of death for the period of 1 January 1996 through 30 November 2004 were acquired from the Office of Economic and Statistical Research of the Queensland Treasury. The causes of all deaths were classified according to the *International Classification of Diseases, 9th Revision* (ICD-9) for 1996, and *10th Revision* (ICD-10) for 1997–2004. The daily counts of nonaccidental deaths (ICD-9: 1–799 and ICD-10: A00–R99), cardiovascular diseases (ICD-9: 390–459 and ICD-10: I00–99), and respiratory diseases (ICD-9: 460–519 and ICD-10: J00–99) were included. Age of death was categorized as all ages, 0–64 years, ≥ 65 years, 65–84 years, and ≥ 85 years.

Environmental data. Daily weather data on maximum, mean, and minimum temperatures and relative humidity (RH) were obtained from the Australia Bureau of Meteorology (http://www.bom.gov.au). Daily air pollution data were received from the Department of Science, Information Technology and Innovation (https://www.qld.gov.au/dsitia/), which included maximum 1-hr average concentrations of ozone (O_3_) and nitrogen dioxide (NO_2_), and 24-hr average concentrations of particulate matter with diameters ≤ 10 μm (PM_10_). The monitoring site for air pollutants and weather conditions was located at the city center.

*Statistical methods*. Model construction. Data analysis was conducted using a Poisson generalized additive model (GAM) allowing for overdispersion to describe the temperature–mortality relationship, because our previous work demonstrated that this model fit the data well (Tong et al. 2012; Yu et al. 2010).

To control for the within-summer seasonal patterns, we used smooth functions of natural cubic splines with 3 degrees of freedom (df) for summer date (i.e., 1–90 for non-leap years or 1–91 for leap years). To avoid overadjustment for between-year variation during the study period, we used a linear function for the year variable. Day of the week and public holidays were also included in the model as dummy variables. Each year’s population data were modeled as an offset to control for the potential confounding effect of demographic shifts over time.

Smoothing functions (natural cubic splines) of temperature parameters (mean, maximum, minimum, and apparent temperatures) with varying lag structures (moving averages of the current and previous days, up to 5 days) were modeled, and the optimal predictor was chosen based on generalized cross-validation (GCV) scores. This procedure was also applied for daily mean relative humidity.

The model is described as follows:

Log [*E*(*Y*)] = α + *ns* (*T_t_*, 3) + *ns* (*RH_t_*, 4) + *ns* (*summer date*, 3) + β*DOW_t_* + γ*year_t_* + δ*Holiday_t_* + *offset* (*population_i_*), [1]

where *t* is the day of death; *E*(*Y*) denotes expected daily death counts; *T_t_* refers to mean temperature (lag 0–1) on day *t*; *RH_t_* is the relative humidity (lag 0–1); *ns* (*…*) refers to natural cubic spline; *DOW_t_* refers to day of the week on day *t*; *year_t_* is an indicator term modeled for each summer which is different from calendar year; *Holiday_t_* is a binary variable that is “1” if day *t* was a holiday; *population_i_* is the population on year *i*; α is the intercept; and β, γ, and δ are coefficients.

This flexible parametric approach was then used to graphically describe relationships between summer temperatures and mortality.

Long-term mortality displacement. To investigate the long-term mortality displacement, we first stratified the summer data (December–February) into two strata according to the previous winter (June–August) mortality levels. To control for confounding by time trend and seasonality, we regressed the time series of daily death counts against time (modeled with natural cubic spline function, 5 df per year). The choice of degrees of freedom was based on comparisons of GCV values. We used the residuals of this model to compute the mean residual values for each winter (μi) separately, as well as for all winters in the whole study period (μw). If the mean residual of a certain year’s winter was higher than the mean value of winters in the whole study period (μi > μw), it was considered as a high-mortality winter, and its following summer was categorized into “H” summer stratum. Otherwise, it was considered as a low-mortality winter (μi < μw), and the following summer was categorized into “L” summer stratum (see Supplemental Material, Table S1). We classified summers separately according to age groups as well as mortality for nonaccidental and CVD deaths. This method was applied only to respiratory mortality for all ages combined because there were insufficient numbers of daily death counts in the age groups. This method had been previously described by Stafoggia et al. (2009).

To estimate the summer temperature effects, we further modelled mean temperature (lag 0–1) with two linear terms constrained to a join point, which is also known as the threshold. Summer temperature-related mortality risks were assumed to be log-linear above the threshold value. The thresholds and their 95% confidence intervals (CIs; 0.1 decimal) were estimated by using the maximum likelihood approach and the resampling technique described in previous studies (Ha et al. 2011; Muggeo and Hajat 2009; Yu et al. 2011). Finally, the integer value of 28°C for mean temperature was chosen as the common threshold (lag 0–1) for all mortality types and age groups, because it fell within the 95% CIs of the thresholds estimated for each age group and disease category.

We estimated summer temperature effects in both the “H” and “L” summer strata and in all summers (for the whole study period), respectively. Then, a dichotomous indicator (HL, “0” for “L” stratum summer days and “1” for “H” stratum summer days) was performed as an interaction term to examine whether the summer heat functions were affected by previous winter mortality levels. The heat slopes and interaction effects above the threshold were also analysed.

This model is described as follows:

Log [*E*(*Y*)] = α + β*_0_T_t_^a^ × HL* + β*_1_T_t_^b^ × HL + ns* (*RH_t_*, 4) + *ns* (*summer date*, 3) + γ*DOW_t_* + δ*year_t_* + ε*Holiday_t_* + *offset* (*population_i_*), [2]

where *T_t_^a^*, *T_t_^b^* refers to mean temperature (lag 0–1) on day *t* above and below the threshold value, respectively; and *HL* represents high or low previous winter mortality.

Short-term mortality displacement. Additionally, short-term lagged effects of summer temperature were investigated using a distributed-lag nonlinear model (DLNM), which is a modeling framework that can simultaneously assess the nonlinear and delayed effects in time-series data (Gasparrini et al. 2010). If there was short-term mortality displacement, negative coefficients of the heat exposures would follow the positive coefficients in the first day(s); the net effect of the heat exposure was estimated by summing coefficients along the lags (Hajat et al. 2005). To capture the main overall temperature effect and adjust for any potential harvesting, we used lags up to 21 days.

The model is described as follows:

Log [*E*(*Y*)] = α + *ns* (*T_t,l_*, 3, 4) + *ns* (*RH_t_*, 4) + *ns* (*summer date*, 3) + β*year_t_* + γ*Holiday_t_* + δ*DOW_t_* + *offset* (*population_i_*), [3]

where *l* is the lag days; *ns* (*T_t,l_*, 3, 4) is a matrix created by 3 df for daily mean temperature and 4 df for lagged effects up to 21 days.

This model was applied to each mortality type in the “H” summer stratum, the “L” summer stratum, and all summers combined, respectively. We estimated the relative risk (RR) of dying on a day with the mean temperature of 29°C compared with 28°C (threshold temperature), and then plotted the RRs against the lags.

*Sensitivity analysis*. We also conducted several sensitivity analyses to check the robustness of our results. First, we classified winters as high- or low-mortality based on the median of the residuals, instead of using the mean, to test the potential influence of model specification on this analysis. Second, we kept the relative differences between previous winter mortality on a continuous scale instead of dichotomizing them, and to check the correlation between annual summer temperature effects and the previous winter mortality levels. The purpose of this analysis was to test whether the effect modification of previous winter mortality was independent of the stratification strategy for the “H” and “L” summer strata. Third, we estimated temperature effects using a linear term for mean temperature (lag 0–1) instead of the piecewise linear functions. Fourth, we added a linear term for the average daily temperature during each summer to determine whether differences in estimated heat effects between H and L summers were influenced by variations in summer temperatures. Fifth, we adjusted for O_3_, NO_2_, and PM_10_ in separate models (modeled as linear terms for lag 0–1) to evaluate potential confounding by air pollution exposures. Finally, we used 5 df for summer date (instead of 3 df) to control for within-summer seasonal patterns.

All statistical analyses were performed using R (R Core Team 2013). The “dlnm” package was used to perform distributed lag nonlinear models (Gasparrini 2011).

## Results

*Descriptive analysis*. Table 1 provides the summary statistics of the daily deaths, weather variables, and air pollutants. There were 53,317 nonaccidental deaths during the study period, with 81.3% of deaths among persons ≥ 65 years old and 32.3% of deaths among those ≥ 85 years old; 42% and 9% died of cardiovascular and respiratory diseases, respectively (Australia Bureau of Statistics 2012). The average daily mean temperature in summer was 24.9°C.

**Table 1:** Summary statistics of climatic variables, air pollutants, and daily deaths in summer, Brisbane, Australia, 1996–2004.

Variable	Minimum	25%	Median	75%	Maximum	Mean ± SD
Mean temperature (°C)	18.3	23.4	24.7	26.3	34.5	24.9 ± 2.3
Maximum temperature (°C)	22.2	27.4	28.5	29.9	40.2	28.7 ± 2.2
Minimum temperature (°C)	14.7	19.1	20.6	22.3	28.1	20.6 ± 2.3
Relative humidity (%)	12	47	52	60	99	55 ± 13.5
O_3_ (ppb)	6	22	28	39	110	32 ± 14.3
NO_2_ (ppb)	5	13	15	18	43	16 ± 5.5
PM_10_ (μg/m^3^)	8	14	17	21	50	18 ± 5.6
Nonaccidental deaths
All ages	5	13	15	18	43	15.5 ± 4.3
0–64 years	0	2	3	4	12	3.0 ± 1.7
≥ 65 years	4	10	12	15	41	12.6 ± 3.9
65–84 years	1	5	7	9	22	7.6 ± 2.9
≥ 85 years	0	3	5	6	23	5.0 ± 2.4
Cardiovascular deaths
All ages	1	5	6	8	31	6.4 ± 2.9
0–64 years	0	0	0	1	4	0.6 ± 0.7
≥ 65 years	0	4	5	7	31	5.8 ± 2.8
65–84 years	0	2	3	4	16	3.1 ± 1.9
≥ 85 years	0	1	2	4	17	2.7 ± 1.8
Respiratory deaths
All ages	0	0	1	2	6	1.2 ± 1.0
0–64 years	0	0	0	0	2	0.1 ± 0.3
≥ 65 years	0	0	1	2	5	1.1 ± 1.0
65–84 years	0	0	0	1	4	0.6 ± 0.8
≥ 85 years	0	0	0	1	4	0.5 ± 0.7
Abbreviations: O_3_, ozone; NO_2_, nitrogen dioxide; PM_10_, particulate matter ≤ 10 μm in aerodynamic diameter. 25% and 75% represent the 25th and 75th percentiles, respectively.						

Table 2 reveals that the average daily mean temperature shows a high variability between winter (15.2°C; range, 14.4–16.4) and summer (24.9°C; range, 23.0–26.2).

**Table 2:** Average daily death counts and temperature in winter and summer seasons in Brisbane, Australia, 1996–2004.

Season/year	All ages	Nonaccidental (age, years)^*a*^				Cardiovascular (age, years)^*b*^					Respiratory^*c*^	Mean temperature (°C)^*d*^
		0–64	≥ 65	65–84	≥ 85	All ages	0–64	≥ 65	65–84	≥ 85		
Winter (June–August)
1996	21.5 (H)	3.6 (H)	17.9 (H)	11.3 (H)	6.6 (H)	10.2 (H)	0.9 (H)	9.3 (H)	5.5 (H)	3.8 (H)	2.1 (H)	15.5
1997	19.9 (L)	3.0 (L)	16.9 (H)	9.9 (L)	6.9 (H)	9.3 (L)	0.7 (H)	8.6 (L)	4.5 (L)	4.1 (H)	1.9 (H)	14.4
1998	19.1 (H)	3.4 (H)	15.7 (H)	9.6 (L)	6.1 (H)	9.1 (H)	0.8 (H)	8.3 (L)	4.7 (L)	3.6 (L)	1.4 (L)	16.4
1999	20.1 (L)	3.2 (L)	16.9 (L)	9.9 (L)	7.0 (L)	9.3 (L)	0.7 (L)	8.6 (L)	4.5 (H)	4.1 (L)	1.9 (H)	15.4
2000	17.9 (L)	2.7 (L)	15.2 (L)	8.3 (L)	6.8 (L)	7.9 (L)	0.6 (L)	7.3 (L)	3.7 (L)	3.7 (L)	1.8 (L)	14.5
2001	18.2 (L)	3.1 (H)	15.1 (L)	8.9 (L)	6.2 (L)	8.0 (L)	0.7 (L)	7.3 (L)	3.8 (H)	3.5 (L)	1.7 (L)	15.1
2002	19.3 (H)	3.1 (H)	16.2 (H)	8.6 (H)	7.6 (L)	8.3 (H)	0.7 (H)	7.7 (H)	3.3 (H)	4.3 (H)	2.0 (L)	14.7
2003	18.2 (H)	3.1 (H)	15.2 (H)	8.2 (H)	6.9 (H)	7.4 (H)	0.6 (L)	6.9 (H)	3.2 (L)	3.6 (H)	2.2 (H)	15.2
2004	17.8 (L)	2.6 (L)	15.3 (L)	8.4 (L)	6.8 (L)	7.0 (L)	0.5 (L)	6.5 (L)	2.9 (L)	3.6 (L)	2.0 (H)	15.4
Summer (December–February)
1996/1997	14.3	3.0	11.3	7.2	4.1	6.0	0.6	5.4	3.2	2.2	1.0	24.4
1997/1998	15.8	3.0	12.8	8.0	4.8	6.9	0.6	6.3	3.6	2.8	1.0	26.2
1998/1999	16.0	2.9	13.1	8.2	4.9	6.8	0.5	6.3	3.5	2.8	1.1	24.7
1999/2000	16.0	2.9	13.0	7.8	5.1	6.6	0.7	5.9	3.1	2.8	1.3	23.0
2000/2001	15.4	3.2	12.2	7.5	4.8	6.2	0.5	5.7	3.1	2.6	1.1	24.6
2001/2002	15.6	2.8	12.8	7.3	5.6	6.2	0.5	5.7	2.8	2.9	1.2	25.7
2002/2003	14.9	3.0	11.9	7.1	4.8	5.9	0.5	5.4	2.9	2.6	1.2	24.6
2003/2004	16.2	2.9	13.3	7.3	5.9	6.4	0.7	5.7	2.8	2.9	1.4	26.1
Abbreviations: H, high previous winter mortality level; L, low previous winter mortality level. ^***a***^Average daily number of nonaccidental deaths. ^***b***^Average daily number of cardiovascular deaths. ^***c***^Average daily number of respiratory deaths. ^***d***^Daily mean temperature on the current day (°C).												

**Figure 1:**
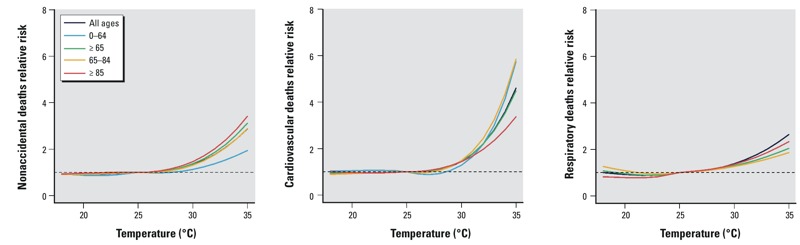
Estimated summer temperature effects (mean temperature, lag 0–1) by age and disease in Brisbane, Australia, 1996–2004.

**Figure 2:**
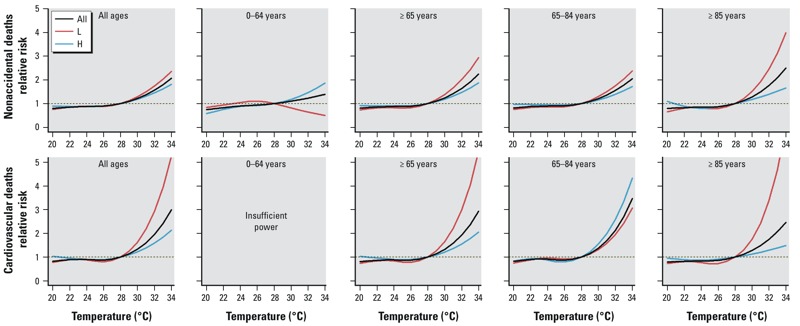
Combined estimated summer temperature effects (mean temperature, lag 0–1) by age and disease for “H” summer stratum (H), “L” summer stratum (L) and for all summers over the whole study period (All) for nonaccidental and CVD mortality.

Figure 1 indicates the J-shaped temperature–mortality relationships in summer. High temperature had a stronger estimated effect on the elderly than the young. Meanwhile, CVD deaths appeared to be more sensitive to high temperature than nonaccidental and respiratory deaths. Estimated effects of high temperature on nonaccidental and cardiovascular mortality were generally stronger in the “L” summer stratum than in the “H” summer stratum (Figure 2), whereas no significant difference between “H” and “L” summers for respiratory deaths was observed (data not shown).

**Figure 3:**
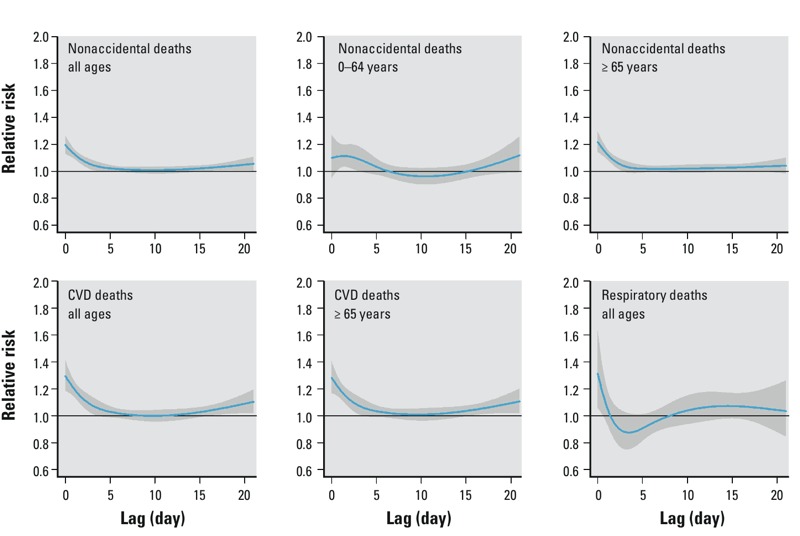
The estimated relative risk of dying on a day with 29°C compared with that on a day with 28°C (threshold temperature) over 21 lagged days for all summers (whole study period).

*Analysis of short-term mortality displacement*. Respiratory mortality was positively associated with summer temperatures above the 28°C threshold on the same day and the following day, but RRs were < 1.0 on lag days 2–8, consistent with short-term mortality displacement (Figure 3). Specifically, the estimated excess risk of respiratory mortality (all ages combined) with a 1°C increase in temperature above the threshold on the same day (lag 0) was 8.84% (95% CI: 2.85, 15.17), whereas cumulative excess risks for lag 0–6 and lag 0–13 were –2.52% (95% CI: –18.65, 16.81) and 5.98% (95% CI: –16.80, 34.99), respectively. Besides, nonaccidental mortality among those 0–64 years of age also showed a negative association with temperature after a 7-day lag, consistent with a weak short-term harvesting effect (Figure 3).

*Examination of the long-term mortality displacement*. Table 3 represents the temperature effects for each summer stratum as well as for all summers in the whole study period. Noticeably, we used 28°C for mean temperature as the common threshold (lag 0–1) for all analyses; however, confidence intervals for the estimated thresholds of each mortality type and age group are also reported in Table 3 to give an idea of the uncertainty around the value of 28°C. Generally, the effect estimates of high temperature on mortality were higher in “L” summer than those in “H” summer. A 1°C increase in mean temperature (lag 0–1) above the threshold was associated with estimated increases in nonaccidental mortality of 11.71% (95% CI: 7.03, 16.60) for “H” summer stratum and 21.58% (95% CI: 13.62, 30.11) for “L” summer stratum in all age groups combined (*p* < 0.01). The difference in excess heat risks between “H” and “L” summer strata was larger in the elderly than all ages. For CVD deaths, a similar pattern was observed, with stronger estimated heat effects in “L” summer. However, results for respiratory deaths were inconsistent. The estimated effects of summer temperature on respiratory mortality were higher in “H” summer than those in “L” summer, although the difference did not reach statistical significance.

**Table 3:** Estimated effects associated with a 1°C increase in summer temperature (mean temperature, lag 0–1) above the threshold by age and diseases in Brisbane, Australia, 1996–2004.

Disease categories/ age groups	Observed threshold^*b*^ (°C)	Percentage increase in mortality above the threshold (95% CI)^*a*^			*p*-Value^*e*^
		All summers	“H” summer^*c*^	“L” summer^*d*^	
Nonaccidental
All ages	28.4 (27.6–28.8)	14.14 (10.15, 18.28)	11.71 (7.03, 16.60)	21.58 (13.62, 30.11)	0.008
0–64 years	30.9 (27.1–32.1)	7.00 (–2.67, 17.63)	9.14 (–1.46, 20.87)	–7.94 (–28.31, 18.22)	0.399
≥ 65 years	27.5 (27.2–28.4)	15.62 (11.15, 20.28)	12.23 (7.12, 17.59)	28.13 (17.86, 39.29)	0.001
65–84 years	27.6 (26.9–28.7)	13.59 (7.46, 20.08)	10.42 (2.30, 9.19)	20.81 (10.00, 32.68)	0.176
≥ 85 years	27.1 (26.7–28.1)	18.18 (11.29, 25.49)	11.70 (3.29, 20.80)	40.10 (24.91, 57.13)	0.000
Cardiovascular
All ages	28.4 (27.3–29.0)	22.29 (15.87, 29.06)	16.55 (8.70, 24.98)	43.23 (30.07, 57.73)	0.000
0–64 years^*f*^	—	—	—	—	—
≥ 65 years	28.4 (27.3–29.1)	22.11 (15.29, 29.33)	15.97 (7.14, 25.54)	45.65 (32.34, 60.31)	0.000
65–84 years	28.8 (27.6–29.4)	25.12 (15.27, 35.82)	43.98 (20.78, 71.62)	20.02 (9.25, 31.86)	0.017
≥ 85 years	28.4 (27.1–28.9)	19.01 (9.45, 29.40)	9.35 (–2.38, 22.49)	57.38 (35.35, 82.99)	0.000
Respiratory
All ages	27.5 (26.2–29.1)	13.00 (–1.39, 29.51)	16.04 (0.64, 33.80)	–23.91 (–54.33, 26.79)	0.108
^***a***^Percentage increase in daily mortality with a 1°C temperature (mean temperature, lag 0–1) increase above the threshold (28°C). ^***b***^Estimated threshold temperature (95% CI) for each mortality type and age group, to show the uncertainty around the common threshold value (28°C). ^***c***^“H” summer stratum, summers with high previous winter mortality. ^***d***^“L” summer stratum, summers with low previous winter mortality. ^***e***^*p*-Value for the interaction term between previous winter mortality levels and summer temperature variable above the threshold from the model. ^***f***^Not estimated due to insufficient death counts.					

**Figure 4:**
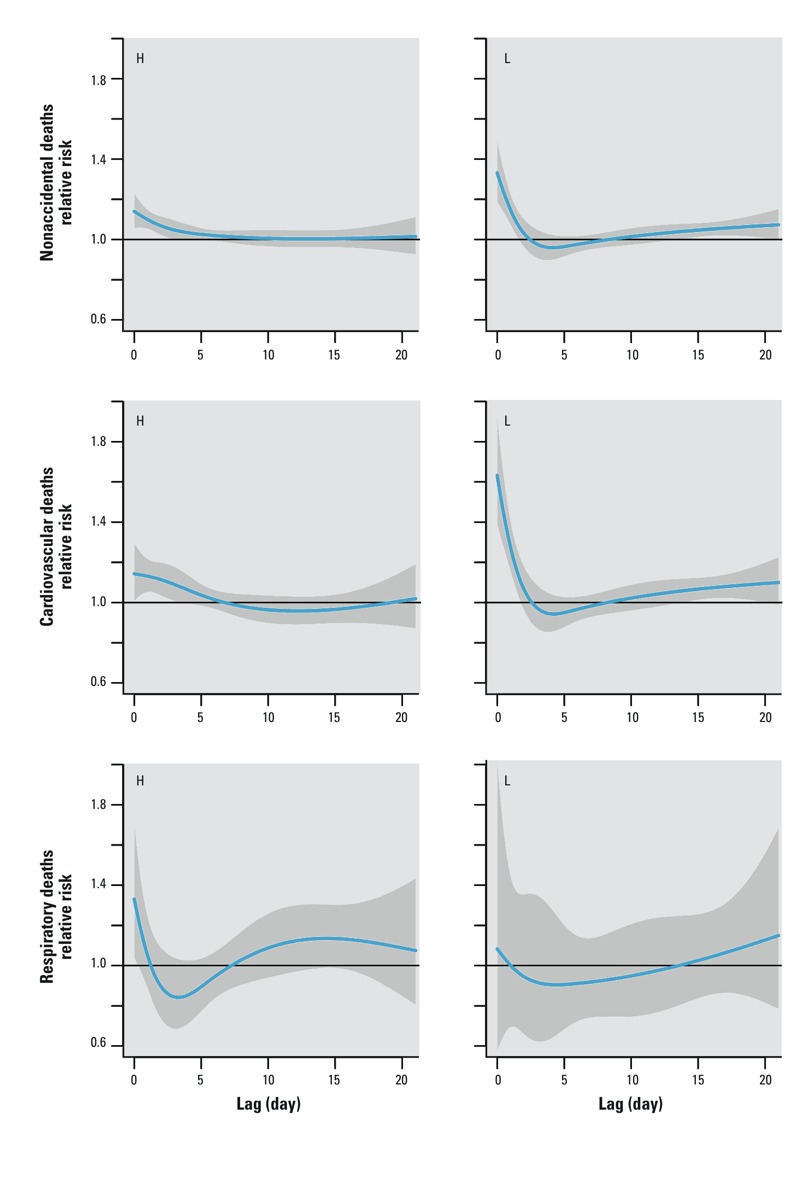
The estimated relative risk of dying on a day with 29°C compared with that on a day with 28°C (threshold temperature) over 21 days of lag by the strata of “H” summer (H) and “L” summer (L).

*Interaction between short-term and long-term mortality displacement*. When we stratified the summers into “L” and “H” strata, a steep decline and an evident deficit appeared after the exposure to high temperatures for nonaccidental and CVD mortality in “L” summer stratum (Figure 4). For example, the RR of dying on a day with 29°C compared with that on a day with 28°C in “L” summer for nonaccidental deaths was 1.33 (95% CI: 1.19, 1.49) on lag day 0, whereas the RR was 0.96 (95% CI: 0.90, 1.02) on lag day 4. The curves also indicate that there were longer lasting heat effects in “H” summer stratum than “L” stratum. For instance, the RRs of dying on a day with 29°C compared with that on a day with 28°C in “L” summer for the nonaccidental mortality were 1.33 (95% CI: 1.19, 1.49), 0.95 (95% CI: 0.89, 1.02), and 0.98 (95% CI: 0.95, 1.02) on lag days 0, 4, and 8, respectively; the corresponding RRs in “H” summer were 1.14 (95% CI: 1.06, 1.23), 1.04 (95% CI: 1.00, 1.08), and 1.01 (95% CI: 0.98, 1.05). Similar patterns were found for other age groups or mortality categories, except for nonaccidental (0–64 years), CVD (65–85 years), and respiratory deaths (see Supplemental Material, Figure S1). The inconsistent result for respiratory deaths might be attributable to the small number of daily death counts.

*Sensitivity analysis*. Classifying winters as high- or low-mortality based on the median value or the mean value of the residuals produced almost identical results (data not shown). When we kept the relative differences between previous winter mortality on a continuous scale, temperature effects on nonaccidental deaths in each summer was inversely associated with the mortality levels in the previous winter. There was the tendency of decreasing heat impacts on nonaccidental mortality in summer as the previous winter mortality increased. When we used a linear term for temperature variable, the pattern of how previous winter mortality modified the summer temperature–mortality relationship remained (see Supplemental Material, Table S2). Overall, the main results changed little when we controlled for air pollution or annual summer’s average temperature (data not shown). When we used 5 df for summer date, the results were similar to our main findings, although the effect estimates were slightly smaller (data not shown).

## Discussion

To the best of our knowledge, this is the first study to investigate both short- and long-term mortality displacement concurrently in heat-related deaths. The major findings of this study are that *a*) there was an apparent effect of high temperatures on mortality in summer, especially among the elderly and for cardiovascular deaths; *b*) there was evidence of short-term mortality displacement for respiratory deaths (all ages combined); and *c*) if the preceding winter mortality level was low, the estimated heat effect for the following summer was stronger, consistent with long-term mortality displacement. This pattern was apparent for nonaccidental and cardiovascular deaths, especially among the elderly; *d*) long-term mortality displacement appeared to affect the short-term mortality displacement for the relationship between temperature and mortality.

Short-term mortality displacement could happen if heat-related deaths mainly occur in chronically ill individuals who will die in a few days or weeks even in the absence of heat exposure (Hajat et al. 2005; Schwartz 2000). Among the limited studies examining short-term mortality displacement, results have been mixed. Some studies reported evidence suggesting that mortality displacement may explain some heat-related deaths (Baccini et al. 2008; Braga et al. 2001; Hajat et al. 2005; Medina-Ramón and Schwartz 2007; Pattenden et al. 2003), but other studies reported that there was little evidence of harvesting effects during extreme heat episodes or on normal hot days (Davis et al. 2004; Kyselý and Kim 2009; Le Tertre et al. 2006; Tong et al. 2010).

The existing evidence suggests that the pattern of mortality displacement may depend on the population at risk (e.g., baseline health status and sociodemographic profile). For example, in our study population, evidence of both short- and long-term mortality displacement was more apparent when the preceding winter mortality was low. Other factors (e.g., behavioral and local factors) might also influence vulnerability (Basu and Malig 2011). Specifically, on a short-term basis, it is believed that harvesting is more likely to occur among the elderly and those already weakened by chronic diseases (Hajat et al. 2005; Schwartz 2000). Furthermore, heat-related mortality displacement has been more strongly associated with deaths from cardiovascular and respiratory diseases than the total nonaccidental deaths in previous studies (Goodman et al. 2004; Hajat et al. 2005).

We also found evidence of short-term mortality displacement for respiratory deaths (all ages combined), as well as evidence of a weak harvesting effect for nonaccidental deaths in the 0- to 64-year age group. Our findings suggest a more immediate and greater degree of short-term mortality displacement for respiratory deaths than CVD deaths. This suggests that deaths that were imminent in individuals with severe respiratory diseases were hastened by exposure to high temperatures, resulting in short-term mortality displacement. However, the situation might differ during heatwaves if individuals with respiratory diseases take extreme care to avoid heat exposure in response to heatwave warnings, thus preventing short-term mortality displacement after a short heatwave event (Le Tertre et al. 2006; Tong et al. 2010). It was a surprise to find evidence of short-term nonaccidental mortality displacement in people < 65 years of age, because short-term mortality displacement is thought to occur mainly among the elderly and frail people. One possible explanation is that people in the work force may be likely to be exposed to heat during summer, and the deaths for those with chronic diseases (even relatively young) might have been brought forward by such an exposure.

In studies of heat-related deaths, long-term mortality displacement may occur if winter deaths are hastened by seasonal risk factors such as cold temperatures or influenza epidemics, thereby reducing the size of the subgroup vulnerable to heat-related mortality in the following summer. As a result, the size of the vulnerable subgroup was reduced (Stafoggia et al. 2009). For instance, a high winter mortality rate among fragile individuals leaves a more “robust” population to face the heat exposure, and therefore mortality in the following summer is likely less than expected. Conversely, low previous winter mortality may inflate the pool of fragile individuals and lead to a larger heat impacts in the summer season.

As far as we know, only three studies have assessed the issue of long-term harvesting effects. Although they all found a modification effect of previous winter mortality on summer temperature-related mortality, the results have not been entirely consistent (Ha et al. 2011; Rocklöv et al. 2009; Stafoggia et al. 2009). Rocklöv et al. (2009) reported that high cardiovascular, respiratory, and influenza mortality in winter was associated with lower estimated effects of temperature on mortality in the following summer, with little evidence of effect modification on total mortality. Stafoggia et al. (2009) focused on the population > 65 years of age. They also reported a higher estimated effect of temperature in summers with lower previous winter mortality, particularly for nonaccidental and CVD deaths, but with relatively weak evidence of mortality displacement for respiratory deaths. Our findings are consistent with the study conducted in Rome, Italy, which has a humid subtropical climate similar to that of Brisbane. We observed evidence of a long-term harvesting effect on nonaccidental deaths for all ages and, to a greater extent, among the elderly. The pattern was similar for CVD deaths, but not for respiratory deaths. However, the lack of evidence for a long-term harvesting effect on respiratory mortality might be attributable to a small number of daily respiratory death counts.

Noticeably, the effect estimates in this study are large compared with those of other studies, perhaps because of the different climatic conditions between different study settings. For example, comparable threshold and similar large heat-related risks have been observed in Palermo, Italy, a city with climatic conditions similar to those of Brisbane (Muggeo and Hajat 2009).

Because the sharing of the pool of frail people, the long-term mortality displacement may also affect the short-term one. For example, after stratified by the previous winter mortality, harvesting in nonaccidental deaths and CVD deaths was only found in the strata of “L” summers. It suggests that low previous winter mortality might inflate the pool of fragile individuals and lead to more immediate heat-related deaths in the following summer. This finding may explain why the observed short-term mortality displacements were heterogeneous in the literature.

This study has several limitations. First, the study was conducted in only one city, so the results need to be interpreted cautiously. Second, the use of data from only one monitoring site may result in exposure misclassification, though a recent study found that single site’s temperatures and averaged temperatures from a network of sites had the similar ability to predict mortality as kriged spatial temperatures in Brisbane (Guo et al. 2012b). Third, due to quite small daily number of influenza deaths, we were unable to evaluate its effect modification in this study. Fourth, we were unable to evaluate confounding by the use of air conditioning.

## Conclusions

We found evidence of an effect of high temperatures on mortality in summer, and evidence of both short- and long-term mortality displacement in the assessment of the heat–mortality relationship in Brisbane, a subtropical city in Australia. These findings may contribute to the knowledge base of understanding the temperature–mortality relationship and assist in developing effective public health intervention measures to reduce and prevent heat-related deaths.

## Supplemental Material

(458 KB) PDF
