# Supplementary material for: Assessment of Short- and Long-Term Mortality Displacement in Heat-Related Deaths in Brisbane, Australia, 1996–2004
Source: Environ Health Perspect. 2015 Mar 20;123(8):766–72. doi: 10.1289/ehp.1307606 (PMC4529002; doi:10.1289/ehp.1307606)
Supplement: (458 KB) PDF [file ehp.1307606.s001.acco.pdf]

Environ Health Perspect
doi: 10.1289/ehp.1307606

Note to Readers: ***EHP*** **strives to ensure that all journal content is accessible to all readers. However, some figures and Supplemental Material published in *EHP* articles may not conform to 508 standards due to the complexity of the information being presented. If you need assistance accessing journal content, please contact ehp508@niehs.nih.gov. Our staff will work with you to assess and meet your accessibility needs within 3 working days.**

Supplemental Material

Assessment of Short- and Long-Term Mortality Displacement in Heat-Related Deaths in Brisbane, Australia, 1996–2004

Zhen Qiao, Yuming Guo, Weiwei Yu, and Shilu Tong

Table of Contents

Table S1. Yearly residuals for each winter and overall mean residual for all winters in the whole study period for non-accidental mortality (all ages combined). Residuals are from the model which regressed the time-series of daily counts of deaths against trend. Provided as an example to clarify how the H/L classification relates to the daily number of deaths.

Figure S1. The estimated relative risk of dying on a day with 29°C compared with a day with 28°C (threshold temperature) over 21 days of lag for the stratum of “H” summer (H) and for “L” summer (L) on each age and mortality categories.

Table S2. Estimated effects associated with 1°C increase in summer temperature (mean temperature, lag 0–1) by age and diseases in Brisbane, Australia, 1996–2004.

Table S1. Yearly residuals for each winter and overall mean residual for all winters in the whole study period for non-accidental mortality (all ages combined). Residuals are from the model which regressed the time-series of daily counts of deaths against trend. Provided as an example to clarify how the H/L classification relates to the daily number of deaths.

Year (winter)	Average daily number of deaths (winter)	Yearly residuals for each winter (μ_i)	Overall mean residual for all winters (μ_w)	Classification of the following summer
1996	21.5	0.071338050	-0.04318664	H
1997	19.9	-0.048803101	-0.04318664	L
1998	19.1	0.008873423	-0.04318664	H
1999	20.1	-0.109256300	-0.04318664	L
2000	17.9	-0.172940006	-0.04318664	L
2001	18.2	-0.080683982	-0.04318664	L
2002	19.3	0.010870957	-0.04318664	H
2003	18.2	0.010710604	-0.04318664	H
2004	17.8	-0.078789381	-0.04318664	L

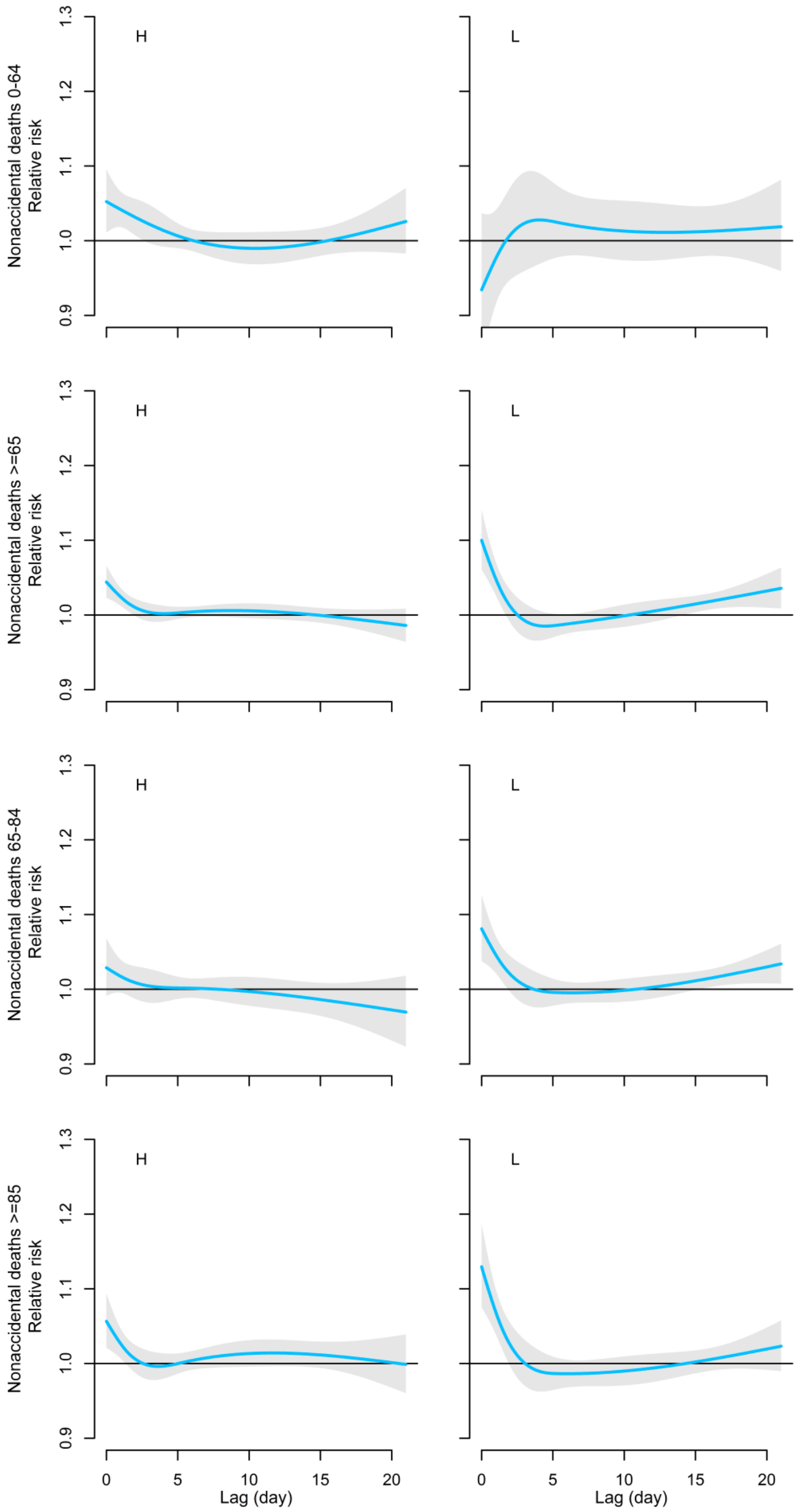

Nonaccidental deaths 0-64
Relative risk
Nonaccidental deaths >=65
Relative risk
Nonaccidental deaths 65-84
Relative risk
Nonaccidental deaths >=85
Relative risk
H
L
0.9
1.0
1.1
1.2
1.3
0
5
10
15
20
Lag (day)

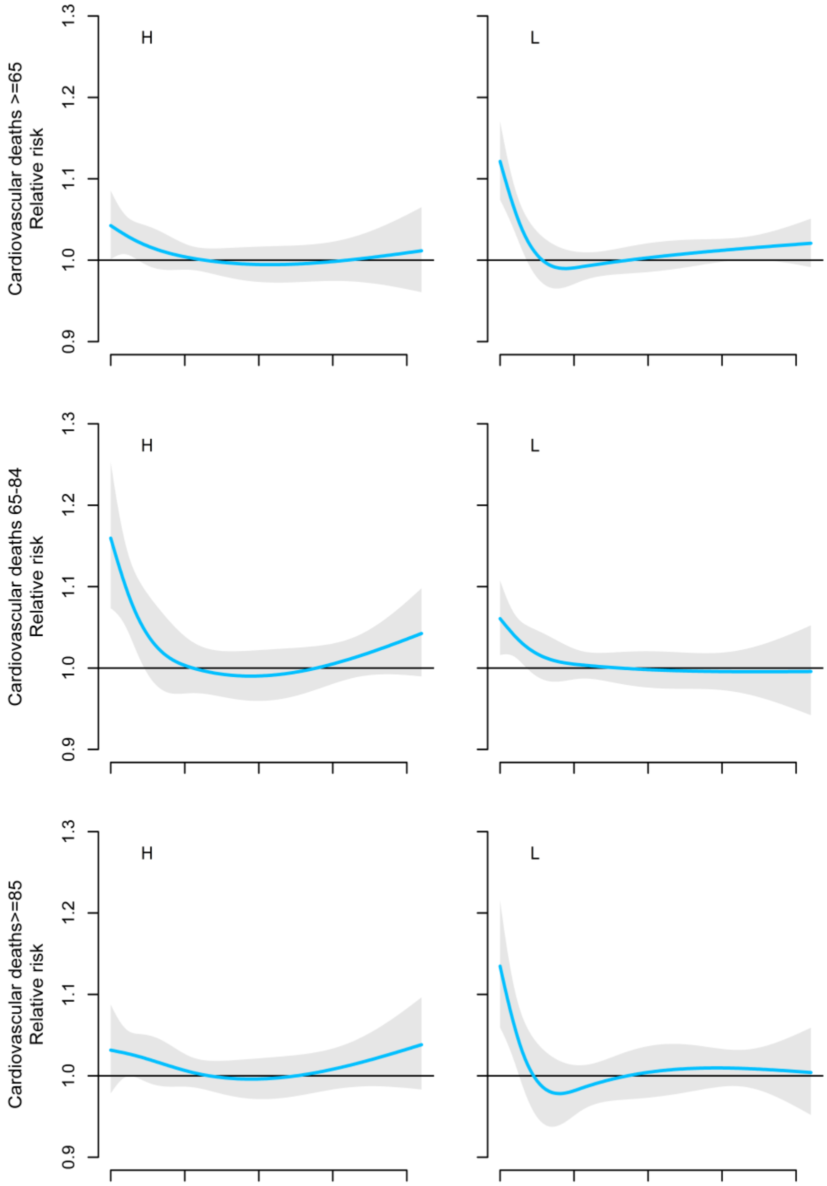

Figure S1. The estimated relative risk of dying on a day with 29°C compared with a day with 28°C (threshold temperature) over 21 days of lag for the stratum of "H" summer (H) and for "L" summer (L) on each age and mortality categories.

Table S2. Estimated effects associated with 1°C increase in summer temperature (mean temperature, lag 0–1) by age and diseases in Brisbane, Australia, 1996–2004.

**Disease categories**	**Age groups**	**Percentage increase in mortality**[a]			***P***[d]
		**All summers**	**"H" summer**[b]	**"L" summer**[c]	
Nonaccidental	All ages	3.88 (2.85, 4.93)	3.64 (2.23, 5.06)	4.01 (2.47, 5.57)	0.710
	0–64 years	3.37 (0.92, 5.87)	4.94 (1.94, 8.02)	0.72 (-3.63, 5.27)	0.070
	≥ 65 years	4.00 (2.83, 5.18)	3.13 (1.63, 4.65)	5.41 (3.49, 7.36)	0.051
	65–84 years	3.39 (1.84, 4.97)	2.20 (-0.31, 4.76)	4.07 (2.07, 6.10)	0.298
	≥ 85 years	4.91 (3.05, 6.81)	4.24 (1.49, 7.06)	5.95 (3.31, 8.65)	0.454
Cardiovascular	all ages	4.09 (2.40, 5.81)	2.86 (0.52, 5.25)	5.20 (2.72, 7.74)	0.098
	0–64 years[e]	—	—	—	—
	≥ 65 years	4.50 (2.71, 6.32)	2.99 (0.24, 5.814)	5.55 (3.15, 8.01)	0.223
	65–84 years	3.92 (1.43, 6.47)	3.77 (0.00, 7.68)	3.89 (0.48, 7.42)	0.861
	≥ 85 years	5.15 (2.54, 7.84)	3.19 (-0.56, 7.09)	7.11 (3.41, 10.94)	0.120
Respiratory	all ages	0.00 (-3.81, 3.96)	2.64 (-1.10, 6.51)	0.00 (-6.56, 7.02)	0.102

[a]Percentage increase in daily mortality with a 1°C temperature (mean temperature, lag 0–1) increase. [b]"H" summer stratum, summers with high previous winter mortality. [c]"L" summer stratum, summers with low previous winter mortality. [d]*p* -Value for the interaction term between previous winter mortality levels and summer temperature variable. [e]Not estimated due to insufficient death counts.
